# Water Absorption, Hydrothermal Expansion, and Thermomechanical Properties of a Vinylester Resin for Fiber-Reinforced Polymer Composites Subjected to Water or Alkaline Solution Immersion

**DOI:** 10.3390/polym11030505

**Published:** 2019-03-16

**Authors:** Xiaoli Yin, Yancong Liu, Yufei Miao, Guijun Xian

**Affiliations:** 1 School of Mechanical and Electronic Engineering, China University of Petroleum, Qingdao 266580, China; yinxl@slcupc.edu.cn (X.Y.); liuyc@upc.edu.cn (Y.L.); 2 Shengli College, China University of Petroleum, Dongying 257061, China; 3 School of Civil Engineering, Harbin Institute of Technology, Huanghe Road No. 73, Nangang District, Harbin 150090, China; miaoyufei@hit.edu.cn

**Keywords:** vinyl ester, durability, water absorption, mechanical properties, immersion

## Abstract

In the present paper, a vinyl ester (VE) resin, potentially used as a resin matrix for fiber-reinforced polymer (FRP) composite sucker rods in oil drilling, FRP bridge cables, or FRP marine structures, was investigated on its resistance to water and alkaline solution immersion in terms of water uptake, hydrothermal expansion, and mechanical properties. A two-stage diffusion model was applied to simulate the water uptake processes. Alkaline solution immersion led to a slightly higher mass loss (approx. 0.4%) compared to water immersion (approx. 0.23%) due to the hydrolysis and leaching of uncured small molecules (e.g., styrene). Water immersion caused the expansion of VE plates monitored with Fiber Bragg Grating (FBG). With the same water uptake, the expansion increased with immersion temperatures, which is attributed to the increased relaxation extent of the resin molecular networks. Although an obvious decrease of the glass transition temperatures (*T*_g_) of VE due to water immersion (5.4 to 6.1 °C/1% water uptake), *T*_g_ can be recovered almost completely after drying. Tensile test results indicate that a short-term immersion (less than 6 months) enhances both the strength and elongation at break, while the extension of the immersion time degrades both the strength and elongation. The modulus of VE shows insensitive to the immersion even at elevated temperatures.

## 1. Introduction

Fiber-reinforced polymer (FRP) composites have been widely accepted as an alternative structural material to steel in oil drilling in recent years [1,2]. Moreover, FRPs find successful applications in construction, e.g., FRP bars for the replacement of steel bars in harsh environments and FRP plates or wet layups for concrete strengthening [3]. The well-recognized advantages of FRPs, such as light weight, corrosion resistance, and high strength, etc., meet the requirements of structural materials in various industries. FRP rods have been successfully applied to various engineering fields, such as sucker rods [4], strengthen/repair existed structures [5], or replace steel bars or cables for the construction of new structures [6]. Despite the excellent corrosion resistance of FRPs in harsh environments, FRPs still face durability problems, which are considered to be a key concern for acceptance in some practice [5,7,8]. The durability of FRPs affects the safe and economic design of FRP structures and needs to be studied comprehensively. As known, compared to the fiber reinforcements, e.g., carbon fibers which are frequently used as reinforcement for FRPs in structural engineering application, the organic resin matrix is considered to be one of the main reasons for the durability problems of FRPs [9,10].

**Figure 1:**
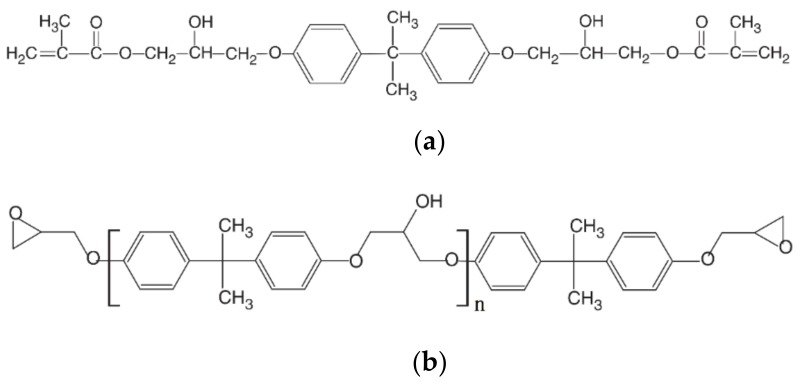
The molecular structures of vinyl ester (VE) (**a**) and commonly used epoxy resin (**b**).

By now, FRPs for structural engineering applications mainly use epoxy and vinyl ester (VE) as matrices. Epoxy resin-based FRPs are more widely used for rods, cables, and wet layups, while VE-based ones are more frequently used for rods or bars produced with a pultrusion process [4,11]. It is worth noting that the VE has a similar molecule backbone (Figure 1), which can be cured with free radicals. For pultrusion, an epoxy resin system is generally cured with anhydride to form three dimensions of molecular structures. Since the formulation of VE includes 30–40 wt % of low molecules, e.g., styrene, the cured VE structures exhibit as less polar compared to the cured epoxy system. As a result, VE shows a high shrinkage due to curing and a lower hydrophilic characteristic [12]. A low hydrophilic characteristic is considered to be beneficial to the hydrothermal ageing resistance; thus, VE resin and its FRPs are more welcomed for marine structures in recent years [13].

In many engineering applications, epoxy resin can be used as adhesives and matrices for all kinds of FRP products and have been well-investigated on their durability [14,15,16,17]. For VE resin, it has been widely used as a resin matrix for glass fiber-reinforced FRPs [18,19]. Some comparative studies on the durability performances of VE and epoxy reinforced FRPs have been conducted recently [20,21,22]. It is worth noting that the resin types show a significant influence on the bonding strength of the fiber and resin, as well as the mechanical properties of FRPs subjected to hydrothermal ageing conditions.

In recent years, with the increasing demands of the high resistance to seawater and the development of suitable sizing for carbon fiber, VE is applied to be used for carbon fiber-FRPs for marine structures [13,23,24]. Generally, VE resin is not considered to be appropriate for carbon fiber-reinforced FRPs due to a poor adhesion to carbon fibers. Both the resin matrix and the fiber–resin bonding are crucial to the mentioned applications.

Although VE is considered to have more and more potentials in various engineering applications (e.g., sucker rods for oil drilling, civil engineering, bridge cables, etc.) exposed to harsh environments, less works have been done on the long-term durability in various conditions compared to the epoxy resin and VE-based FRPs. In view of this, the present paper aims to investigate the durability of VE resin immersed in a water or alkaline solution in terms of the water absorption, mechanical properties, and dynamic thermal mechanical properties. The changes of the properties of the VE will be compared to those of an epoxy resin. The study will help understand the durability of VE-based FRPs and will benefit the acceptance of VE in various structural applications encountering harsh environments.

## 2. Materials and Methods

### 2.1. Raw Materials

**Figure 2:**
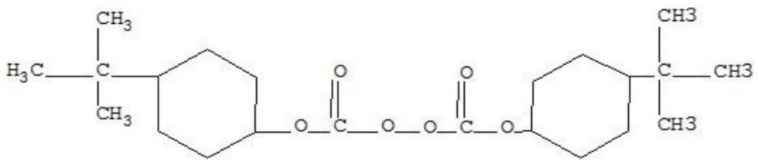
The molecular structure of Perkadox-16.

VE resin used in the present study was a kind bisphenol-A epoxy-based vinyl ester resin (MFE-2) produced by Sino Polymer Co. Ltd. (Shanghai, China). The initiator was tert-butyl hydroperoxide (TBHP), and the accelerator was Perkadox-16 with the molecular structure shown in Figure 2. The initiator and accelerator were also provided by Sino Polymer Co. Ltd. The mixture ratio of VE:TBHP:Perkadox-16 was 100:1:0.2 by weight.

The datasheet of the VE resin provided by the producer had a solid content around 57.0 ± 3 wt %, indicating the styrene content was about 40 wt %.

### 2.2. Sample Preparation

The mixture of the VE resin system (including VE resin, initiator, and accelerator) in the given ratio was cured in an open aluminum mold with a cavity of 200 mm × 150 mm × 4 mm. The curing process occurred in an oven; followed the procedure 50 °C for 1 h and then 80 °C for 1 h, 100 °C for 1 h, and 140 °C for 1 h; and then was cooled down to the room temperature in the oven.

The cured VE plate was then cut into designed dimensions with a water cooling saw for further tests.

### 2.3. Water Uptake Test

The VE samples for water uptake were cut into the dimensions of 25 mm × 25 mm × 4 mm. The surface of the samples was carefully polished with fine sandpapers and then conditioned in an oven at 60 °C for two days before immersion.

The immersion media were chosen as distilled water and an alkaline solution. The alkaline solution was used to simulate the concrete pore water with a pH value around 12.5. The alkaline solution was prepared according to Reference [25]. The resin samples were immersed in water or alkaline solution baths at 20 °C, 40 °C, and 60 °C for 6 months. After a predetermined period, the samples were removed from the bath, dried with tissue papers, and weighed with an electric balance with an accuracy of 0.01 mg. The water uptake was calculated with the following equation.

(1) $$W(\%)=\frac{W_{t}-W_{0}}{W_{0}}\times 100\%$$

where *W* (%) was the weight gain, *W_t_* was the weight at time *t*, and *W_0_* was the dry weight at *t* = 0.

For each immersion condition, 10 samples were tested and the average data was reported.

### 2.4. Sample Drying

Samples for water uptake, mechanical property, and dynamic mechanical thermal analysis (DMTA) immersed for 3 or 6 months were taken out from the immersion bath and dried in an oven at 60 °C. The weights of the water uptake samples were measured periodically. The drying process was terminated when the variation of the last five weight points was less than 5%.

### 2.5. Characterization

The details of the internal strain tested with FBG sensors can be found in our previous work [26]. It should be noted that we calibrated the reported strain in this work by changing the temperatures.

DMTA test (Q800, TA Instruments, New Castle, USA) was conducted on VE specimens with a size of 40 mm × 8 mm × 4 mm. The DMTA tests were performed with the tensile mode, 1 Hz frequency heated from 25 °C and 200 °C at 5 °C/min.

The tensile properties of the VE specimens with 4 mm of thickness were tested according to ASTM D 638–2010 (American Society for Testing Materials, Standard Test Methods for Tensile Properties of Plastics). The type of specimens was chosen as the Type IV specimen. The speed of testing was 5 mm/min. For each condition, five samples were repeated and the average results were reported (the variance among different specimens was the standard deviation with reference to ASTM D 638–2010).

The flexural properties of VE plates (4 mm in thickness) were evaluated under the three-point bending mode using a Universal Testing Machine (DHY-10080, Hengyi Precision Instrument Co. Ltd., Shanghai, China) in accordance with ASTM D 790. The samples were cut per the ASTM standard. The machine was operated at a crosshead speed of 1 mm/min, and a support span-to-depth ratio of 16:1 was used. At least five specimens were tested for each type of composite, and the average value was reported.

## 3. Results and Discussion

### 3.1. Water Uptake and Diffusion

**Figure 3:**
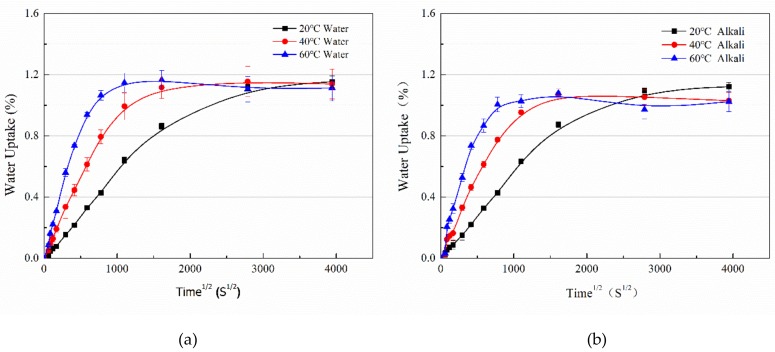
The water uptake versus the square root of time for VE specimens exposed to 20 °C, 40 °C, and 60 °C water (**a**) or an alkaline solution (**b**).

Figure 3 shows the water uptake versus the square root of the immersion time at 20 °C, 40 °C, and 60 °C in distilled water (Figure 3a) or an alkaline solution (Figure 3b) for half of a year. Each discontinuous point represented the average weight gain for ten samples and was obtained by Equation (1).

As shown in Figure 3, after half of a year of immersion, all specimens for both the immersion conditions reached saturation status. To obtain the water uptake and diffusion parameters, the curve fitting method was adopted with two-stage water uptake models [27]:

(2) $$M_{t}=M_{\infty }\left(1+k\sqrt{t}\right)\left\{1-\exp\left[-7.3{\left(\frac{Dt}{h^{2}}\right)}^{0.75}\right]\right\}$$

where *M_∞_* means saturation water uptake, *k* is the relaxation related constant, *t* is the immersion time in second, *D* is the water diffusion constant, and *h* is the thickness of the samples.

It is worth noting that the constant *k* can be considered related to both the relaxation of the resin network (positive *k*) and the hydrolysis of the resin or leaching of uncured small molecules, i.e., styrene (negative *k*), during immersion [27]. The smaller the value of *k* is, the higher the extent of the hydrolysis or leaching. With the curve fitting method using Equation (2) according to Reference [27], the water diffusion parameters were determined and are summarized in Table 1.

As shown in Table 1, for the water immersion, increasing the immersion temperature leads to a bit of an increase in the saturation water uptake (*M_∞_*). On the contrary, for the alkaline solution immersion, *M_∞_* decreases with the immersion temperature slightly. As mentioned above, the contradictory effects of the relaxation of resin networks and hydrolysis/leaching on the water uptake determines the final saturated water uptake. For the alkaline solution immersion, hydrolysis/leaching occurred, which was supported by the evolution of *k* and the drying test (see the following section). Hydrolysis/leaching led some parts of the resin or uncured styrene to be diffused into the solvent, resulting in the mass reduction. For the water immersion, hydrolysis/leaching was much less, and the relaxation of the resin network, which tended to absorb more water molecules, contributed to the increased water uptake.

The hydrolysis rate of the resin system can be compared with the constant of *k* [27]. Accordingly, immersed in water, VE resin shows an obvious hydrolysis/leaching at 60 °C. At this temperature, *k* is negative, meaning an occurrence of mass loss. However, for the alkaline solution immersion, the hydrolysis becomes obvious even at 40 °C. An increase of the immersion temperature to 60 °C for the immersion in the alkaline solution, the hydrolysis becomes more pronounced with a smaller value of *k*. It is worth noting that the hydrolysis of cured VE networks is expected to be coming from the existence of the ester groups (see in Figure 1). Alkaline exhibits a catalyzing effect, being responsible for the higher hydrolysis.

Undoubtedly, *D* increases with the immersion temperatures for both immersion cases (Table 1). Compared with the epoxy resin systems [28,29] and polyurethane resin [9], *D*_s_ of the current VE resin is located in the ranges of 10^−6^ mm^2^/s to 10^−5^ mm^2^/s. The activation energy (*E_a_*) of *D*_s_ for both immersion cases can be determined according to Reference [9]. For the water immersion, *E_a_* was equal to 53.4 kJ/mol, while for the alkaline solution immersion, *E_a_* was reduced to 49.6 kJ/mol. Both *E_a_*s were in the range of values of epoxy and polyurethane, which may indicate the water diffusion mechanisms were similar for these three kinds of resins. The slightly reduced *E_a_* for the alkaline immersion case might have been attributed to the serious hydrolysis of the networks. In this case, the water diffusion paths in the hydrolyzed networks may have encounter less hindering.

Table 2 presents the mass loss of the immersed VE resin samples for 3 or 6 months. Clearly, as also mentioned above, the alkaline solution immersion brings in a higher mass loss compared to the water immersion, and the increased immersion temperatures leads to more mass loss. The enhanced hydrolysis of the resin system due to the catalyzing effect of the alkaline solution and the acceleration effect of a high temperature is responsible for the trends.

In addition, the longer immersion period leads to the higher mass loss for both immersion cases. However, the mass loss after 3 or 6 months of immersion at 60 °C is very close (0.23% to 0.24% for water immersion and 0.39% to 0.42% for alkaline solution immersion). The trend indicates that the hydrolysis/leaching levels off. Compared to the water immersion, the alkaline solution tends to catalyze the hydrolysis of the ester groups, and a more serious mass loss occurrs.

### 3.2. Hygrothermal Expansion

The expansion of the VE plate during immersion was tracked with FBG. Due to the corrosion of the optical fiber exposed to a strong alkaline solution, only water-immersed VE samples were investigated in the present study.

**Figure 4:**
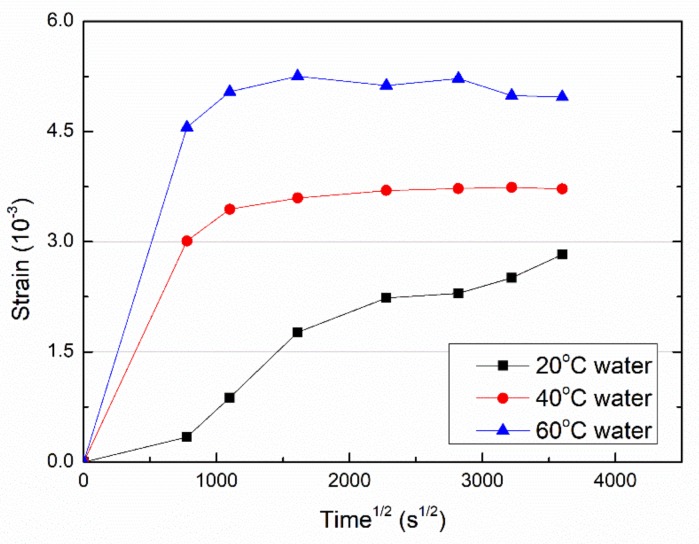
The strain of the VE plate immersed in water as a function of immersion time.

Since the expansion of the VE plate is expected to be coming from the water uptake, the *x*-axis of the expansion curve (Figure 4) is set as the square root of immersion time to a facilitate comparison with the water uptake curve (Figure 1a).

Compared to Figure 1a, the evolution of the strain vs. square root of immersion time (Figure 4) is similar to the water uptake curve: an initial fast increase and then levelling off. However, the strain is not directly related to the water uptake. The higher immersion temperature leads to a higher expansion strain (Figure 4). For example, the maximum strain is around 5% and 3.7% for 40 and 60 °C water immersion, while the saturated water uptake is closed, around 1.1% (Table 1). In addition, for 20 °C water immersion, the strain still increases rather than levels off (Figure 4), and the maximum strain reaches 2.8% after 150 days immersion.

The higher strain due to a higher temperature water immersion is attributed to the enhanced relaxation of the resin network. At increased temperatures, the water molecules may diffuse into dense molecular networks and leads the dense network to expand. At low temperatures, the water molecules tend to stay in loose network regions and will not bring in less expansion.

### 3.3. Glass Transition Temperatures

**Figure 5:**
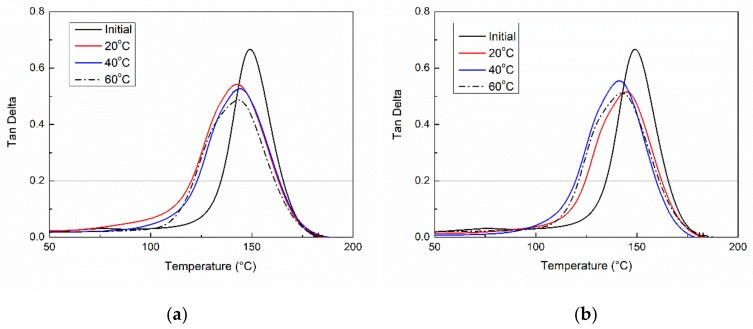
The tan δ curves of the VE samples subjected to water immersion (**a**) or an alkaline solution (**b**) at various temperatures for 6 months.

Figure 5 presents the tan δ curves of the initial VE samples and ones immersed in water or the alkaline solutions for 6 months at 20 °C, 40 °C, and 60 °C. The glass transition temperatures (*T_g_*s) were determined by the peak of the tan δ curves. After 6 months of immersion, the *T_g_* of the VE samples reduced from 149.3 °C to 142.5 °C, 144.2 °C, and 143.2 °C for water immersion and to 144.9 °C, 140.5 °C, and 142.8 °C for the alkaline solution immersion at 20 °C, 40 °C, and 60 °C, respectively. The reduction of *T_g_*s is undoubtedly attributed to the plasticization of the absorbed water [29]. According to the saturated water uptake (see in Table 1), the decrease of *T_g_* is around 5.4 °C/1% water uptake for the water immersion and 6.1 °C/1% water uptake for the alkaline solution immersion.

In comparison to the initial VE samples, the tan δ curves of the immersed VE samples became wider and dwarfed due to the ununiform distribution of water molecules and also its plasticization effect [30].

**Figure 6:**
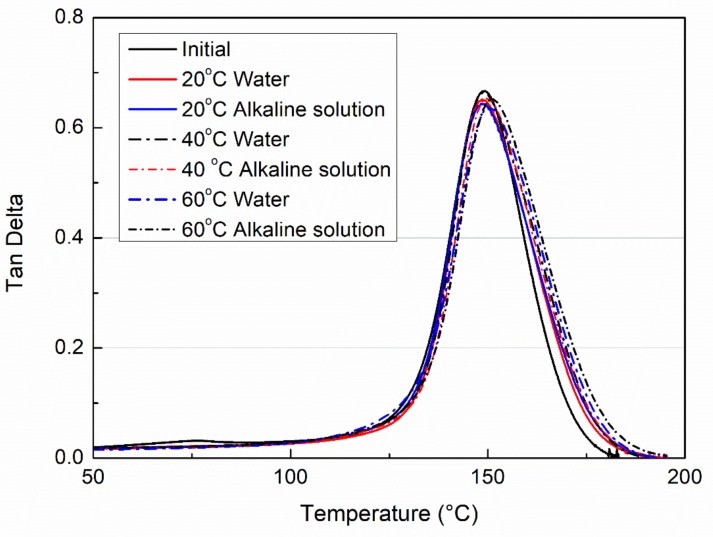
The tan delta curves of the dried VE samples which were subjected to water immersion or alkaline solution for 6 months.

Figure 6 presents the tan δ curves of the dried VE samples which were immersed in water or an alkaline solution for 6 months. As seen, all curves of the initial sample and immersed samples are almost completely overlapped. This indicates that most of the immersion effects are reversible. Although hydrolysis in the case of the alkaline solution immersion or a leaching of small uncured molecules occurred as mentioned above, such irreversible effects may mainly occur on the sample surface. Especially since the immersion time only lasts 6 months, such effects were not significant. As reported, with an increase of times, an immersion in water or alkaline will lead to the formation of multi-bound water molecules and affects the viscoelastic properties of the resin system [30].

### 3.4. Evolution of Mechanical Properties

**Figure 7:**
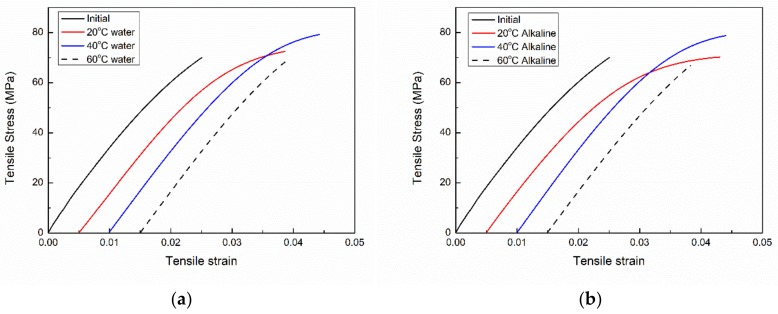
The tensile stress vs. strain curves of the VE samples which were subjected to water (**a**) or an alkaline solution (**b**) immersion for 6 months.

Figure 7 presents tensile stress vs. strain curves of the initial and immersed VE samples for 6 months. For the un-aged VE plate, the strain–stress curve shows a brittle feature, and the strain–stress curve is almost linear. The tensile strength and the elongation at break are 70 MPa and 2.49%, respectively.

With a short-term immersion (i.e., less than 6 months) in both water and alkaline solution, the VE plates show an increase in both the tensile strength and elongation at break. For example, after a 4-month immersion, the tensile strength and elongation at break increased to 79.2 MPa and 3.4% for the water immersion and 78.7 MPa and 3.4% for the alkaline solution immersion. VE subjected to a short-term of immersion shows an enhanced nonlinearity (Figure 7). Plasticization due to water ingress is expected to be responsible for this enhancement.

After 6 months of immersion, the VE plates exhibit similar strain–stress curve shapes to the initial VE ones with a slight decrease in both the strength and elongation at break (Figure 7). As expected, hydrolysis at elevated temperatures as well as in the alkaline solution mainly happens on the surfaces of the VE plates. This brings in some defects on the surfaces of the plates, leading to a reduced strength.

It is worth noting that not much was different for the immersions in alkaline solution and water immersion on the tensile curves. The immersion time only lasts 6 months, and the probably formed defects on the surfaces of plate samples does not affect the whole tensile properties of the plates.

It is worth noting that the slopes of the initial linear part of the strain–strain curves are almost parallel for the unaged and aged VE samples (Figure 7a,b). This indicates the tensile modulus (approx. 3.5 GPa) is insignificantly affected by the immersion.

**Figure 8:**
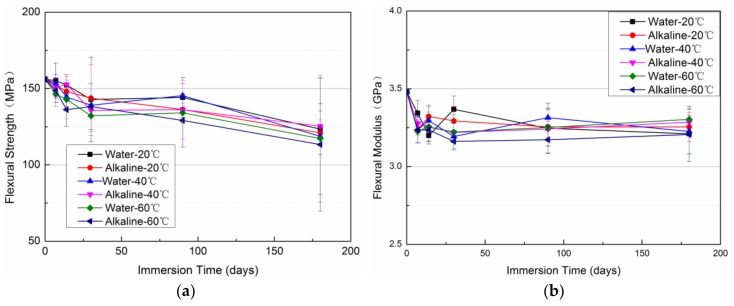
The flexural strength (**a**) and modulus (**b**) of the VE samples vs. immersion time.

The flexural properties of the un-aged and aged VE plates were also investigated. The evolution of the flexural strength and modulus are given in Figure 8a,b. Not many different effects between the alkaline solution immersion and water immersion on the flexural properties are seen. This is similar to the tensile properties. In addition, flexural strength decreases slowly with immersion time, while the flexural modulus levels off, insensitive to the immersion time.

## 4. Conclusions

In the current work, a VE resin system used for fiber-reinforced polymer composites for sucker rods or bridge cables was investigated on its resistance to water and alkaline solution immersion. The water uptake, hydrothermal expansion, and thermal and mechanical properties were studied as a function of immersion time for 6 months. The following conclusions can be drawn based on the study.

(1) Immersion in an alkaline solution causes a mass loss (approx. 0.4%) due to hydrolysis and the leaching of uncured small molecules.
(2) With the same water uptake, the expansion of VE samples increased with the immersion temperatures, which is attributed to the increased relaxation extent of the resin molecular network.
(3) Immersion in both water and an alkaline solution leads to a remarkable decrease of the glass transition temperatures of the VE samples, while they can be recovered almost completely by drying.
(4) The tensile strength and elongation at break can be enhanced for a short term (less than 6 months) of immersion but will be degraded by an extended immersion period.

## Figures and Tables

**Table 1:** The water uptake, diffusion coefficients, and *k* of VE resin immersed in distilled water or an alkaline solution.

Immersion Media	Temp. (°C)	*M_∞_* (%)	*K* (10^−6^)	D (10^−6^ mm^2^/s)
Water	20	1.07	21.41	0.78
	40	1.11	8.54	2.84
	60	1.15	−9.57	8.70
Alkaline solution	20	1.11	3.09	0.77
	40	1.08	−9.96	2.63
	60	1.05	−11.15	7.24

**Table 2:** The mass loss (%) of the immersed VE samples for 3 or 6 months.

Immerse Conditions	Mass Loss (%) at		
	20 °C	40 °C	60 °C
3 months water	0.07	0.11	0.23
3 months Alkaline	0.13	0.15	0.39
6 months water	0.09	0.19	0.24
6 months Alkaline	0.17	0.31	0.42
